# SOCIAL INEQUITIES AND THE IMPAIRMENT PARADOX IN LATER LIFE

**DOI:** 10.1093/geroni/igad104.0804

**Published:** 2023-12-21

**Authors:** Michal Engelman, Heide Jackson

**Affiliations:** University of Wisconsin-Madison, Madison, Wisconsin, United States; University of Maryland, College Park, Maryland, United States

## Abstract

Research on health across the life course consistently documents widening racial and socioeconomic disparities from childhood through adulthood, followed by stabilization or convergence in later life. This pattern appears to contradict expectations set by cumulative (dis)advantage (CAD) theory. Informed by the punctuated equilibrium perspective, we examine the relationship between midlife health and subsequent health change and mortality and consider the impact of earlier socioeconomic exposures on observed disparities. Using the Health and Retirement Study, we characterize the functional impairment histories of a nationally representative sample of 8464 older adults between 1994 and 2016. We employ nonparametric and discrete outcome multinomial logistic regression to examine the competing risks of mortality, health change, and attrition. As expected, exposures to disadvantages are associated with poorer functional health in midlife and mortality. However, we find that a higher number of functional limitations in midlife is negatively associated with the accumulation of subsequent limitations for White men and women and for Black women, and the impact of educational attainment, occupation, wealth, and marriage on later-life health differs across race and gender groups. We conclude that the observed stability or convergence in later-life functional health disparities is not a departure from the dynamics posited by CAD, but rather a result of the differential impact of racial and socioeconomic inequities on mortality and health at older ages. Higher exposure to disadvantages and a lower protective impact of advantageous exposures lead to higher mortality among Black Americans, a pattern which masks persistent health inequities later in life.

